# Bearing-based localization for leader-follower formation control

**DOI:** 10.1371/journal.pone.0175378

**Published:** 2017-04-20

**Authors:** Qing Han, Shan Ren, Hao Lang, Changliang Zhang

**Affiliations:** 1 Engineering College, Honghe University, Honghe Yunnan, China; 2 School of Mechanical Engineering, Northwestern Polytechnical University, Xi’an, China; Peking University, CHINA

## Abstract

The observability of the leader robot system and the leader-follower formation control are studied. First, the nonlinear observability is studied for when the leader robot observes landmarks. Second, the system is shown to be completely observable when the leader robot observes two different landmarks. When the leader robot system is observable, multi-robots can rapidly form and maintain a formation based on the bearing-only information that the follower robots observe from the leader robot. Finally, simulations confirm the effectiveness of the proposed formation control.

## Introduction

Multi-robot formation control has long been a topic of interest in both academic research and industrial applications. Furthermore, the advantages of multi-robot systems over a single robot include greater flexibility, adaptability and robustness [1–3]. Based on these characteristics, typical applications for multi-robot formations include underwater or outer space exploration, shop floor transportation, guarding, escorting, airport snow shoveling, surveillance and patrolling missions [4–7]. However, multi-robot formation control is challenging, especially when the observation information is poor and the system is highly nonlinear. A variety of formation control methods have been proposed, such as the virtual structure approach [8,9], the behavior-based approach [10], the leader-follower approach [11–14], the artificial potential approach [15] and the graph theory approach [16]. Among these approaches, the leader-follower approach has been the most widely used method owing to its simplicity, scalability and reliability. However, the majority of existing leader-follower approaches require the distance-angle information or more information [13].

In real environments, the available observations of multi-robot systems could be the only bearing observations, which pose significant challenges for formation control. If the leader robot can observe no less than two different landmarks while the robot and the landmarks are not collinear, then the system is completely observable [16]. In [17], the observability analysis of cooperative locations for two robots is presented with bearing-only measurements, and the robot states are not observable with respect to a global reference frame. It has also been shown that two landmarks are needed for the observability of a single vehicle [18–20]. The leader robot plays a critical role in the robot formation control. In previous studies, the trajectories of the leader robot for multi-robot formation control had to be ideal. When the leader robot system is observable, the ideal trajectories of the leader robot for formation control will be avoided, and the leader-follower formation can run along complex trajectories and maintain the desired formation. Only a few special cases regarding bearing-only formation control problems have been solved. In [21–23], bearing-only control laws that can guarantee global stability for the formation control of only three or four robots are presented. In [24], observability conditions for position tracking are established based on bearing-only observations, and the localization problem is studied using the extended output Jacobian. To our knowledge, bearing-only leader-follower formation control for multi-robots that do not require the trajectories of the leader robot to be ideal is still a relatively new research topic that has not attracted much attention; thus, this topic is the focus of the present study.

To restrict multi-robot formation errors, the system must be observable, and the estimation techniques must be used to solve the localization problem. Estimation techniques include the unscented Kalman filter (UKF) [11], the extended information filter (EIF) [16], the particle filter (PF) [25] and the extended Kalman filter (EKF) [26].

Our approach in this paper can be applied to situations with n robots. This paper offers two main contributions. First, to achieve multi-robot formation control, a PF estimation algorithm is used to solve the leader robot localization problem. Furthermore, we ensure that the leader robot system is completely observable if the leader robot can observe no less than two different landmarks. In this case, the trajectories of the leader robot for formation control are not required to be ideal. Second, control of the leader-follower formation for multi-robots is studied based on the bearing-only UKF algorithm and input-output feedback control to achieve rapid multi-robot formation that can remain consistent.

The rest of this paper is organized as follows. In Section II, the leader robot localization problem based on the bearing-only observation is presented. Section III presents leader-follower formation control for multi-robots based on the bearing-only UKF algorithm and input-output feedback control. Simulation results are given in Section IV, and in Section V, we offer our conclusions.

## Leader robot localization based on bearing-only observations

### Modeling

**Fig 1:**
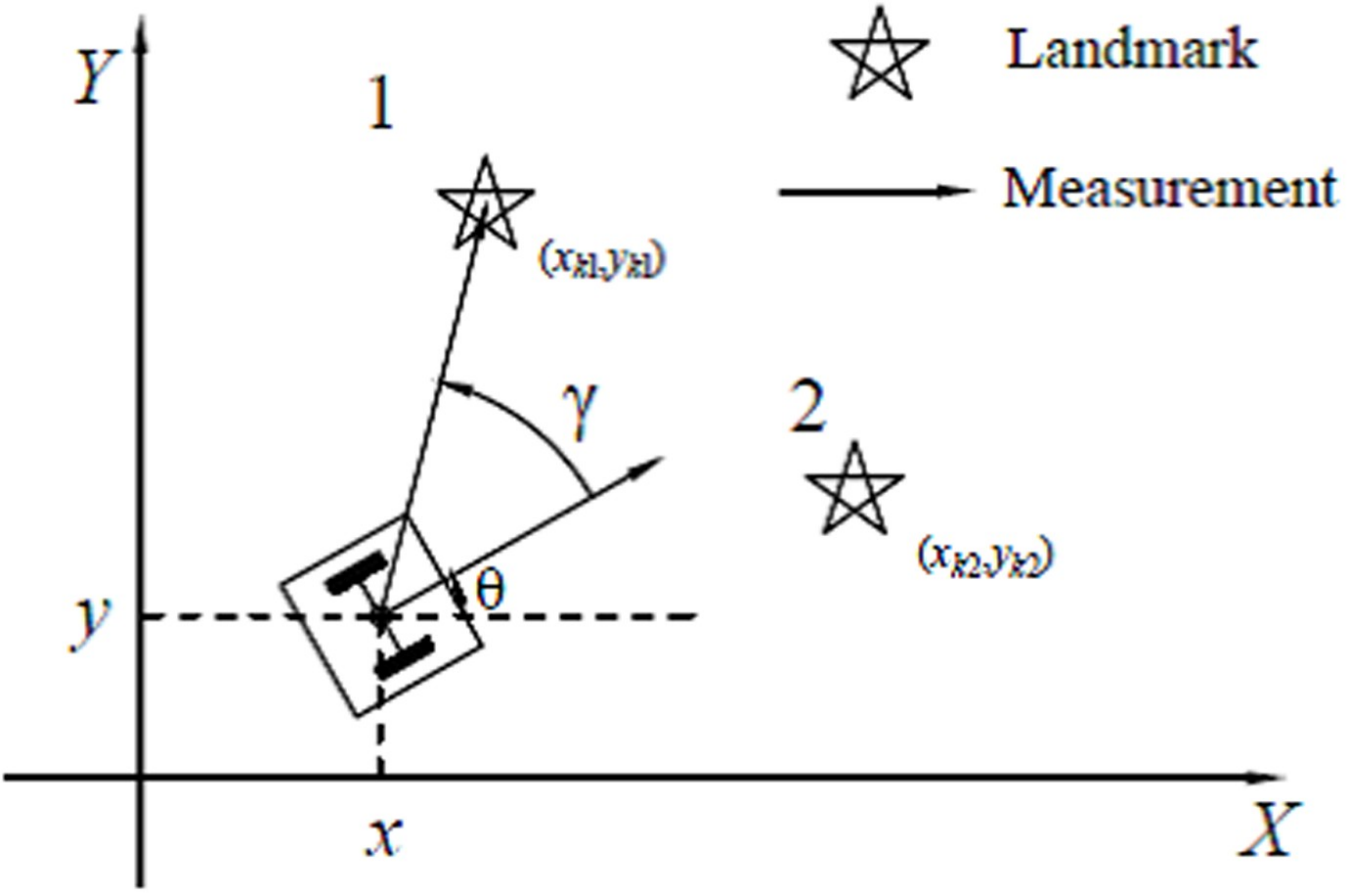
Relative bearing measurements of the leader robot with two different landmarks.

The setup considered throughout this paper is for *n* robots. The *i*th robot kinematics can be abstracted as

$${\dot{S}}_{i}=f_{i\left(S_{i},u_{i}\right)}\underline{\underline{⊳}}\left(\begin{array}{c} \nu_{i}\text{cos}\theta_{i}\\ \nu_{i}\text{sin}\theta_{i}\\ \omega_{i} \end{array}\right)$$ (1)

where *s*_*i*_ = [*x*_*i*_ *y*_*i*_ *θ*_*i*_]^T^ ∈ ℜ^3^ is the robot state, including the robot position [*x*_*i*_ *y*_*i*_] and the robot orientation *θ*_*i*_, and *U*_*i*_ = [*v*_*i*_ *ω*_*i*_]^T^ is the control input. Without loss of generality, we assume that robots can only move forward (*v*_*i*_ ≥ 0, *i* = 1,⋯,*n*). Each robot uses the exteroceptive sensors to measure its bearings relative to the other robots and the known landmarks that are in the field of view of the sensors. As shown in Fig 1, the leader robot can obtain bearing measurements by measuring two different landmarks, and relative bearings from the *i*th robot to the *j*th robot or landmark can be written as

$$\gamma_{ij}=\text{arctan}\left(\frac{y_{j}-y_{i}}{x_{j}-x_{i}}\right)-\theta_{i}$$ (2)

### Observability analysis of the leader robot system

The PF method is used to determine the location of the leader robot based on bearing-only information and to approximate the state of the leader robot; the position estimation of the leader robot is critical to improving the localization accuracy. The leader robot plays a crucial role in the leader-follower multi-robot formation control. When the leader robot system is observable, the formation errors are bounded, and the leader robot’s trajectories are very close to the true trajectories. Based on the nonlinear observability rank criteria [27], we derive the linearly independent rows in the observability matrix for the leader robot that observes a certain number of landmarks. To compute the Lie derivatives, the nonlinear kinematic equation in Eq (1) is changed into the following convenient form:

$${\dot{s}}_{i}=f_{\nu_{i}}\nu_{i}+f_{w_{i}}\omega_{i}$$ (3)

The zeroth-order Lie derivative of any scalar function is the function itself, i.e.,

$$L_{}^{0}h_{k}(s,z)=h_{k}(s,z)$$ (4)

The first-order Lie derivative of the function *h*_*k*_ (*s*, *z*) with respect to $f_{\nu_{i}}$ is defined as

$$L_{f_{\nu }{}_{{}_{i}}}^{1}h_{k}(s,z)=\nabla L_{}^{0}h_{k}(s,z)\cdot f_{\nu_{i}}$$ (5)

Here, ∇ represents the gradient operation and · denotes the vector inner product.

Because $L_{f_{\nu }{}_{{}_{i}}}^{1}h_{k}(s,z)$ is a scalar function, the second-order Lie derivative of *h*_*k*_ (*s*, *z*) with respect to $f_{\nu_{i}}$ is

$$L_{f_{\nu }{}_{{}_{i}}f_{\nu }{}_{{}_{i}}}^{2}h_{k}(s,z)=\nabla L_{f_{\nu }{}_{{}_{i}}}^{1}h_{k}(s,z)\cdot f_{\nu_{i}}$$ (6)

Higher order Lie derivatives are computed similarly. Additionally, mixed Lie derivatives are defined. The second-order Lie derivative of *h*_*k*_ (*s*, *z*) with respect to $f_{\nu_{j}}$, given its first derivative with respect to $f_{\nu_{i}}$, is

$$L_{f_{\nu }{}_{{}_{i}}f_{\nu }{}_{{}_{j}}}^{2}h_{k}(s,z)=\nabla L_{f_{\nu }{}_{{}_{i}}}^{1}h_{k}(s,z)\cdot f_{\nu_{j}}$$ (7)

Based on the preceding expressions for the Lie derivatives, the observability matrix is defined as

$$M=\left[\nabla L_{f_{\nu }{}_{{}_{i}},\cdots ,f_{\nu }{}_{{}_{j}},f_{\omega_{i}},\cdots f_{\omega_{j}}}^{p}h_{k}\left(s,z\right)\right]$$ (8)

where *i*, *j* = (1,⋯,*n*), *k* = (1,⋯,*m*) and *p* ∈ N. The important role of this matrix in the observability analysis of a nonlinear system is demonstrated by Theorem 1.

**Theorem 1:** A system is locally weakly observable if its observability matrix, *M*, has full rank, e.g., rank (*M*) = 3*n* in our case.

The necessary Lie derivatives of *h*_*k*_ (*s*, *z*) and the corresponding gradients are computed, and the observability matrix, *M*, is obtained. In the first case, the observability condition that a robot observes a landmark is analyzed.

For simplicity, we assume that $c\theta_{i}\underline{\underline{⊳}}\text{cos}\theta_{i}$ and $s\theta_{i}\underline{\underline{⊳}}\text{sin}\theta_{i}$. In Eq (3),

$$f_{\nu_{i}}={\left[\begin{array}{ccc} c\theta_{i} & s\theta_{i} & 0 \end{array}\right]}^{\text{T}}$$ (9)

$$f_{\omega_{i}}=[\begin{array}{ccc} 0 & 0 & 1 \end{array}{]}^{\text{T}}$$ (10)

The zeroth-order Lie derivative is

$$L_{}^{0}h_{k}(s,z)=\gamma_{ik_{1}}=\text{arctan}\left(\frac{y_{k_{1}}-y_{i}}{x_{k_{1}}-x_{i}}\right)-\theta_{i}$$ (11)

and its gradient scaled by $R_{{}_{ik_{1}}}^{2}$ is given by

$$\nabla L^{0}h_{k}\left(s,z\right)=\left[\begin{array}{ccc} -\Delta y_{ik_{1}} & \Delta x_{ik_{1}} & -R_{{}_{ik_{1}}}^{2} \end{array}\right]$$ (12)

where $\Delta x_{ik_{1}}=x_{i}-x_{k_{1}}$, $\Delta y_{ik_{1}}=y_{i}-y_{k_{1}}$, and $R_{{}_{ik_{1}}}^{2}={\left(\Delta x_{ik_{1}}\right)}^{2}+{\left(\Delta y_{ik_{1}}\right)}^{2}$.

The first-order Lie derivatives are

$$L_{{}^{f_{v_{i}}}}^{1}h_{k}\left(s,z\right)=\Delta x_{ik_{1}}s\theta_{i}-\Delta y_{ik_{1}}c\theta_{i}$$ (13)

$$L_{f_{\omega }{}_{{}_{i}}}^{1}h_{k}(s,z)=-R_{ik_{1}}^{2}$$ (14)

and their gradients are given by

$$\nabla L_{{}^{f_{v_{i}}}}^{1}h_{k}\left(s,z\right)=\left[\begin{array}{ccc} \text{s}\theta_{i} & -\text{c}\theta_{i} & \Delta x_{ik_{1}}\text{c}\theta_{i}+\Delta y_{ik_{1}}\text{s}\theta_{i} \end{array}\right]$$ (15)

$$\nabla L_{{}^{f_{\omega_{{}_{i}}}}}^{1}h_{k}\left(s,z\right)=2\left[\begin{array}{ccc} -\Delta x_{ik_{1}} & -\Delta y_{ik_{1}} & 0 \end{array}\right]$$ (16)

Thus, the gradients of the second-order and higher order Lie derivatives are linearly dependent on the rows of the observability matrix corresponding to the gradients of the first-order and zeroth-order Lie derivatives. Therefore, we can write the observability matrix with the rows corresponding to $\gamma_{ik_{1}}$ using the gradients of the Lie derivatives up to the first order as follows:

$$M_{ik_{1}}=\left[\begin{array}{ccc} -\Delta y_{ik_{1}} & \Delta x_{ik_{1}} & -R_{{}_{ik_{1}}}^{2}\\ \text{s}\theta_{i} & -\text{c}\theta_{i} & \Delta x_{ik_{1}}\text{c}\theta_{i}+\Delta y_{ik_{1}}\text{s}\theta_{i}\\ -2\Delta x_{ik_{1}} & -2\Delta y_{ik_{1}} & 0 \end{array}\right]$$ (17)

**Lemma 1:** The rank of the observability matrix, $M_{ik_{1}}$, given by Eq (17) is 2 if

1. *V_i_* > 0, *i* = 1,⋯,*n*; and
2. the robot does not move along the line that joins the robot and the landmark.

**Proof:** If the robot does not move along the line that joins the robot and the landmark, the $M_{ik_{1}}$ matrix in Eq (17) can be transformed into the simplified form shown in Eq (18) using a finite sequence of elementary row operations.

$$M_{ik_{1}}\Rightarrow \left[\begin{array}{ccc} 1 & 0 & \Delta y_{ik_{1}}\\ 0 & 1 & -\Delta x_{ik_{1}}\\ 0 & 0 & 0 \end{array}\right]$$ (18)

The two nonzero rows in Eq (18) are linearly independent. Therefore, the rank of Eq (18) is 2, i.e., rank $\left(M_{ik_{1}}\right)=2$. Therefore, from Theorem 1, the single robot states are not locally weakly observable.

Similarly, the observabilities of a robot that observes two different landmarks is also analyzed. The rank of the observability matrix can be easily obtained using the same method as that in Lemma 1.

$f_{v_{i}}$ and $f_{\omega_{i}}$ are the same as in Eqs (9) and (10), respectively. Because a robot observes two different landmarks, *h*_*k*_ (*s*, *z*) is defined as

$$h_{k}(s,z)=\left[\begin{array}{c} \gamma_{ik_{1}}\\ \gamma_{ik_{2}} \end{array}\right]$$ (19)

The zeroth-order Lie derivative is

$$L_{}^{0}h_{k}(s,z)=\left[\begin{array}{c} \gamma_{ik_{1}}\\ \gamma_{ik_{2}} \end{array}\right]$$ (20)

and its gradient scaled by $\left[\begin{array}{cc} R_{ik_{1}}^{2} & 0\\ 0 & R_{ik_{2}}^{2} \end{array}\right]$ is given by

$$\nabla L^{0}h_{k}\left(s,z\right)=\left[\begin{array}{ccc} -\Delta y_{ik_{1}} & \Delta x_{ik_{1}} & -R_{{}_{ik_{1}}}^{2}\\ -\Delta y_{ik_{2}} & \Delta x_{ik_{2}} & -R_{{}_{ik_{2}}}^{2} \end{array}\right]$$ (21)

where, $\Delta x_{ik_{1}}=x_{i}-x_{k_{1}}$, $\Delta x_{ik2}=x_{i}-x_{k_{2}}$, $\Delta y_{ik_{1}}=y_{i}-y_{k_{1}}$, $\Delta y_{ik_{2}}=y_{i}-y_{k_{2}}$, $R_{{}_{ik_{1}}}^{2}={\left(\Delta x_{ik_{1}}\right)}^{2}+{\left(\Delta y_{ik_{1}}\right)}^{2}$ and $R_{{}_{ik_{2}}}^{2}={\left(\Delta x_{ik_{2}}\right)}^{2}+{\left(\Delta y_{ik_{2}}\right)}^{2}$.

The first-order Lie derivatives are

$$L_{{}^{f_{v_{i}}}}^{1}h_{k}\left(s,z\right)=\left[\begin{array}{c} \Delta x_{ik_{1}}\text{s}\theta_{i}-\Delta y_{ik_{1}}\text{c}\theta_{i}\\ \Delta x_{ik_{2}}\text{s}\theta_{i}-\Delta y_{ik_{2}}\text{c}\theta_{i} \end{array}\right]$$ (22)

$$L_{f_{\omega }{}_{{}_{i}}}^{1}h_{k}(s,z)=\left[\begin{array}{c} -R_{ik_{1}}^{2}\\ -R_{ik_{2}}^{2} \end{array}\right]$$ (23)

and their gradients are given by

$$\nabla L_{{}^{f_{v_{i}}}}^{1}h_{k}\left(s,z\right)=\left[\begin{array}{ccc} \text{s}\theta_{i} & -\text{c}\theta_{i} & \Delta x_{ik_{1}}\text{c}\theta_{i}+\Delta y_{ik_{1}}\text{s}\theta_{i}\\ \text{s}\theta_{i} & -\text{c}\theta_{i} & \Delta x_{ik_{2}}\text{c}\theta_{i}+\Delta y_{ik_{2}}\text{s}\theta_{i} \end{array}\right]$$ (24)

$$\nabla L_{{}^{f_{\omega_{{}_{i}}}}}^{1}h_{k}\left(s,z\right)=\left[\begin{array}{ccc} -2\Delta x_{ik_{1}} & -2\Delta y_{ik_{1}} & 0\\ -2\Delta x_{ik_{1}} & -2\Delta x_{ik_{1}} & 0 \end{array}\right]$$ (25)

We can write the observability matrix with the rows corresponding to $\left[\begin{array}{c} \gamma_{ik_{1}}\\ \gamma_{ik_{2}} \end{array}\right]$ using the gradients of the Lie derivatives up to the first order as shown in Eq (26).

$$M_{ik_{12}}=\left[\begin{array}{ccc} -\Delta y_{ik_{1}} & \Delta x_{ik_{1}} & -R_{{}_{ik_{1}}}^{2}\\ -\Delta y_{ik_{2}} & \Delta x_{ik_{2}} & -R_{{}_{ik_{2}}}^{2}\\ \text{s}\theta_{i} & -\text{c}\theta_{i} & \Delta x_{ik_{1}}\text{c}\theta_{i}+\Delta y_{ik_{1}}\text{s}\theta_{i}\\ \text{s}\theta_{i} & -\text{c}\theta_{i} & \Delta x_{ik_{2}}\text{c}\theta_{i}+\Delta y_{ik_{2}}\text{s}\theta_{i}\\ -2\Delta x_{ik_{1}} & -2\Delta y_{ik_{1}} & 0\\ -2\Delta x_{ik_{2}} & -2\Delta y_{ik_{2}} & 0 \end{array}\right]$$ (26)

**Lemma 2:** The rank of the observability matrix given by Eq (26) is 3 if

1. *V_i_* > 0, *i* = 1,⋯,*n*;
2. the robot does not move along the line that joins the robot and the landmark; and
3. the robot and two landmarks are not collinear (i.e., $\gamma_{ik_{1}}\neq \gamma_{ik_{2}}$).

**Proof:** To meet the three prerequisites above, the $M_{ik_{12}}$ matrix in Eq (26) can be transformed into the simplified form shown in Eq (27) using a finite sequence of elementary row operations.

$$M_{ik_{12}}\Rightarrow \left[\begin{array}{ccc} 1 & 0 & 0\\ 0 & 1 & 0\\ 0 & 0 & 1\\ & 0_{3\times 3} & \end{array}\right]$$ (27)

The results show that the simplified form of the $M_{ik_{12}}$ matrix has three linearly independent rows, i.e., rank $\left(M_{ik_{12}}\right)=3$. Therefore, from Theorem 1, the single robot states are completely observable.

From Lemma 2, we know that if all of the *n* robots in the group can directly observe two different landmarks, then the system is completely observable (i.e., rank (*M*) = 3*n*). If all of the *n* robots in the group can directly observe more than two different landmarks, the system can obtain more observation information and is also more observable. If a system is locally weakly observable, the system output can convey sufficient information to allow the observer to provide a correct estimate of the state, thus effectively improving the localization of the leader robot. In a non-observable system, the output does not convey sufficient information to allow the observer to provide a correct estimate of the state, thus negatively affecting the localization of the leader robot.

## Leader-follower formation control for multi-robots based on the bearing-only UKF algorithm and input-output feedback control

### Problem statement

**Fig 2:**
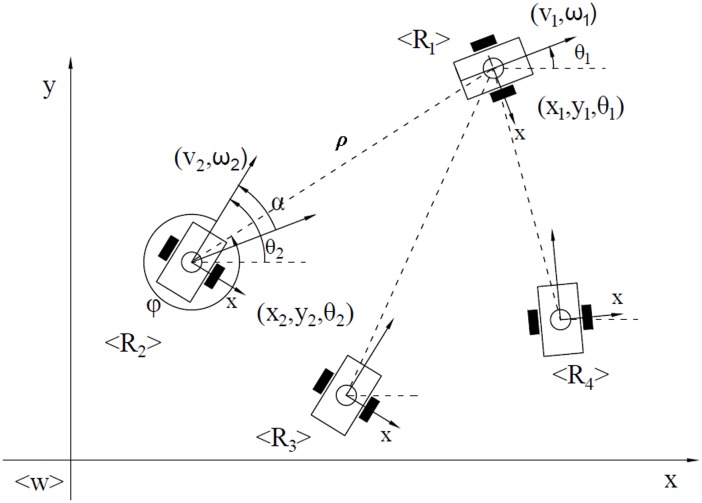
Leader-follower coordinate representation.

Based on the observabilities of a leader robot that observes two landmarks, the bearing-only leader-follower formation control is studied in this section. We formulate the leader-follower formation model and define the notations used. As shown in Fig 2, *R*_1_ represents the leader robot, while *R*_2_, *R*_3_ and *R*_4_ are the follower robots. The control inputs for the robots are the linear and angular velocities [*v*_*i*_ *ω*_*i*_]^T^, where (*i* = 1,⋯,*n*). *ρ*_*i*_ is the distance from the centroid of the leader to the centroid of the follower, and *φ*_*i*_ is the view angle from the y-axis of the follower robot to the centroid of the leader robot. *θ*_*i*_ and *θ*_*j*_ are the orientations of the leader robot and the follower robot with respect to the world frame 〈W〉, respectively, and *α*_*i*_ is the relative orientation between the leader robot and the follower robot, i.e.,$\alpha_{i}\underline{\underline{\Delta }}\theta_{i}-\theta_{j}$.

With reference to Fig 2, the kinematic model of a formation with one leader and one follower in a polar coordinate system can be readily defined as shown in Eq (28). The kinematic model of a formation with one leader and *n* followers can be defined as shown in Eq (29).

$$S_{n}:\{\begin{array}{c} \dot{s}=f(s,u)={\left[\begin{array}{cc} f_{1}^{\text{T}}\left(s_{1},u_{1}\right), & f_{2}^{\text{T}}\left(s_{1},u_{2}\right) \end{array}\right]}^{\text{T}}=F(s)U\\ y=h(s)={\left[h_{1}^{\text{T}}(s)\right]}^{\text{T}}={\left[\begin{array}{cc} \varphi_{1} & \alpha_{1} \end{array}\right]}^{\text{T}} \end{array}$$ (28)

where the state vector is $s\underline{\underline{\Delta }}{\left[s_{1}^{\text{T}}\right]}^{\text{T}}$, $s\underline{\underline{\Delta }}{\left[\begin{array}{ccc} \rho_{1} & \varphi_{1} & \alpha_{1} \end{array}\right]}^{\text{T}}$, *δ*_1_ = *φ*_1_ + *α*_1_, the input vector is $U\underline{\underline{\Delta }}\left[\begin{array}{cccc} \nu_{1} & \omega_{1} & \nu_{2} & \omega_{2} \end{array}\right]$, the output vector is $y=h(s)={\left[h_{1}^{\text{T}}(s)\right]}^{\text{T}}$, $h_{1}\underline{\underline{\Delta }}\left[\begin{array}{cc} \varphi_{1} & \alpha_{1} \end{array}\right]$ and $F(s)=\left[\begin{array}{cccc} \text{c}\delta_{1} & 0 & -\text{c}\varphi_{1} & 0\\ -\text{s}\delta_{1}/\rho_{1} & 0 & \text{s}\varphi_{1}/\rho_{1} & -1\\ 0 & 1 & 0 & -1 \end{array}\right]$.

$$S_{n}:\{\begin{array}{c} \dot{s}=f(s,u)={\left[\begin{array}{ccc} f_{1}^{\text{T}}\left(s_{1},u_{1}\right), & \ldots , & f_{n+1}^{\text{T}}\left(s_{n},u_{n\text{+}1}\right) \end{array}\right]}^{\text{T}}\\ y=h(s)={\left[\begin{array}{ccc} h_{1}^{\text{T}}\left(s_{1}\right), & \ldots , & h_{n}^{\text{T}}\left(s_{n}\right) \end{array}\right]}^{\text{T}} \end{array}$$ (29)

### Input-output feedback control based on bearing-only UKF

**Fig 3:**
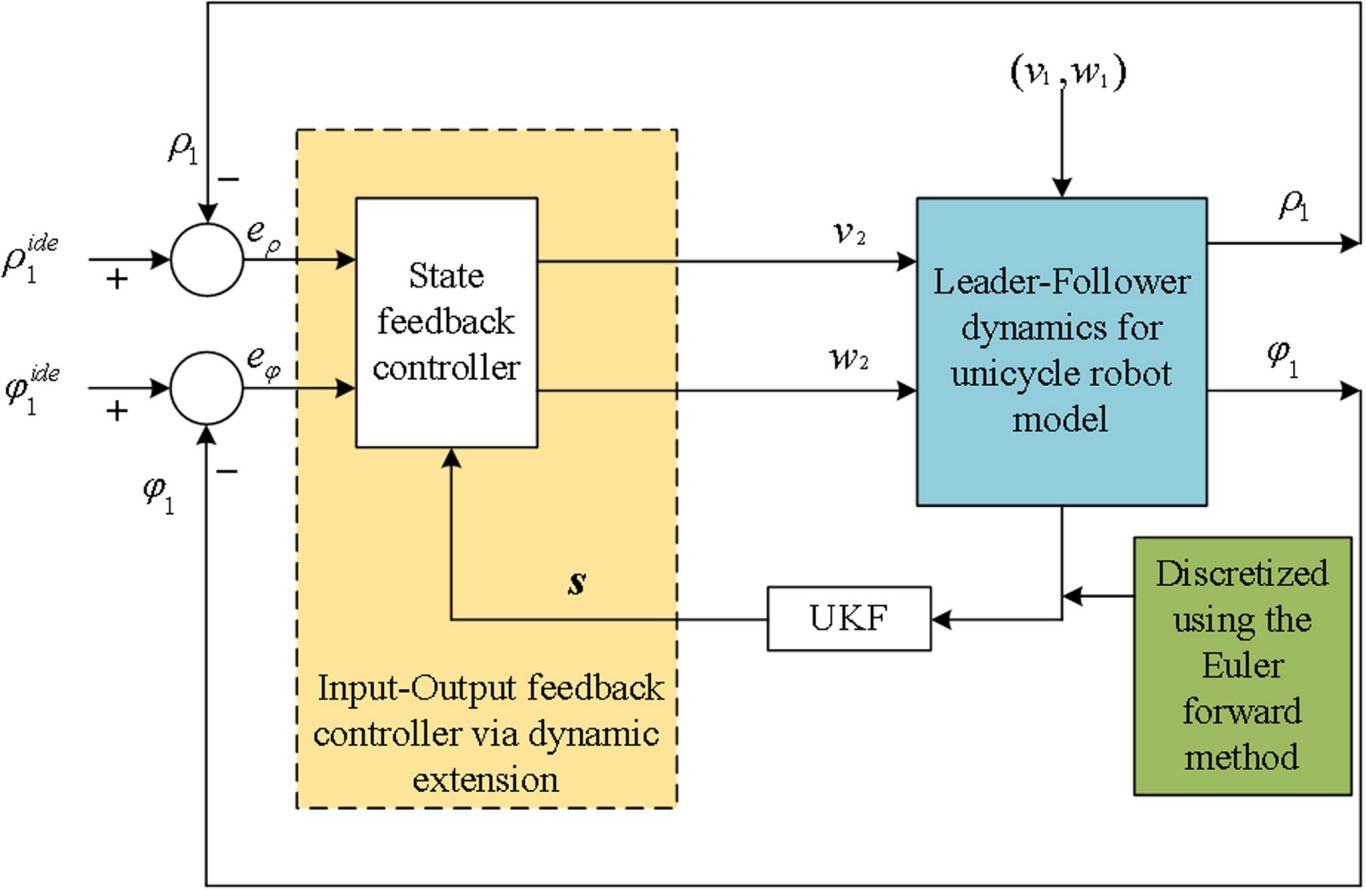
Block diagram of the input-output feedback control based on the UKF.

To achieve and maintain the desired leader-follower formation, the follower robots require information regarding the relative position of the leader robot to adjust their positions in real time. In our approach, the leader robot position is estimated using UKF, and the follower positions are calculated using the classical input-output feedback control law; this system is shown Fig 3. In the following, we explain how to achieve input-output feedback control based on the bearing-only UKF.

The variable $\hat{s}$ is the state estimation of the true state ***s***. The UKF is designed to estimate the angle information, i.e., [*φ*_*i*_ *α*_*i*_]^T^ of the state ***s*** given the input, *U*, and the output, ***y***. Assuming that both the state and the observation equations are affected by additive noises, we obtain

$$\dot{s}=F(s)U\text{+O}$$ (30)

y=Cs+N (31)

where *C* is the output transition matrix and *O* and *N* are white Gaussian noises with zero mean and zero covariance matrices *P*_*O*_ and *P*_*N*_, respectively. For simplicity, we assume that ***s***(0) and that *O* and *N* are uncorrelated, and then we apply the Euler forward method with a sampling time *T*_*c*_, to discretize the state dynamics in Eq (30), so Eq (32) is obtained.

$$s(k+1)=\Gamma (s(k),u(k))+T_{c}O$$ (32)

where $\Gamma (s(k),u(k))=T_{c}F(s)U+s(k)$ and *k* ∈ N.

In [11], the UKF is based on the unscented transformation, which includes a prediction and a correction step. To improve the robot localization, the process equation in Eq (30) and the observation equation in Eq (31) must be sampled.

The control law is designed for *R*_1_ and *R*_2_, similar to the design for the other robots of a multi-robot formation. Changing the form of the state equation in Eq (28), the reduced state-space kinematic model of the multi-robot formation is equivalent to Eqs (33) and (34) is obtained by taking the time derivative of $\alpha_{1}\underline{\underline{\Delta }}\theta_{1}-\theta_{2}$,

s˙1rΔ__[ρ˙1φ˙1]T=Fr(s1)U=[cosδ10−cosφ10−sinδ1ρ10sinφ1ρ1−1][ν1ω1ν2ω2]=[cosδ10−sinδ1ρ10][ν1ω1]+[−cosφ10sinφ1ρ1−1][ν2ω2]=L(s1)U1+M(s1)U2 (33)

$${\dot{\alpha }}_{1}=\omega_{1}-\omega_{2}$$ (34)

where $s_{1r}\underline{\underline{\Delta }}{\left[\begin{array}{cc} \rho_{1} & \varphi_{1} \end{array}\right]}^{\text{T}}$ is the reduced state-space vector and the matrices $L{\left(s_{1}\right)}^{2\times 2}=\left[\begin{array}{cc} \text{cos}\delta_{1} & 0\\ -\text{sin}\delta_{1}/\rho_{1} & 0 \end{array}\right]$ and $M{\left(s_{1}\right)}^{2\times 2}=\left[\begin{array}{cc} \text{-cos}\varphi_{1} & 0\\ \text{sin}\varphi_{1}/\rho_{1} & -1 \end{array}\right]$ are the two upper-left and right submatrices of *F*_*r*_(***s***_1_), respectively.

Using the standard techniques of input-output linearization in [28], we propose an input-output state feedback control for multi-robot formation control. According to Eq (33), we consider the following control input for *R*_2_:

$$U_{2}\underline{\underline{\Delta }}{\left[\begin{array}{cc} \nu_{2} & \omega_{2} \end{array}\right]}^{\text{T}}=M^{-1}\left(s_{1}\right)\left(C-L\left(s_{1}\right)U_{1}\right)$$ (35)

where $C=-K(s_{1r}-s_{1r}^{\text{ide}}),\;K=diag\left[\begin{array}{cc} k_{1} & k_{2} \end{array}\right]$, with *k*_1_, *k*_2_ > 0. The superscript “ide” refers to the desired state, and *C* is the auxiliary control parameter. Eqs (34) and (35) act as a feedback linearizing control in Eq (33), so that the closed-loop dynamics are shown in Eq (36).

$$\begin{array}{l} {\dot{s}}_{1r}=C=-K(s_{1r}-s_{1r}^{\text{ide}})=\left[\begin{array}{c} k_{1}(\rho_{1}^{\text{ide}}-\rho_{{}_{1}}^{})\\ k_{2}(\varphi_{1}^{\text{ide}}-\varphi_{{}_{1}}^{}) \end{array}\right]\\ {\dot{\alpha }}_{1}=\omega_{1}-\omega_{2} \end{array}$$ (36)

## Simulation study

**Fig 4:**
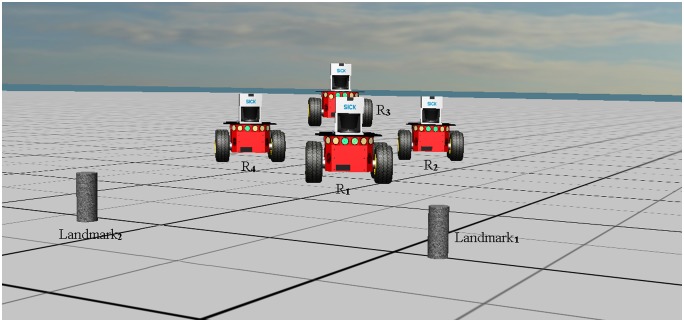
The simulation scene of the mobile robots.

**Fig 5:**
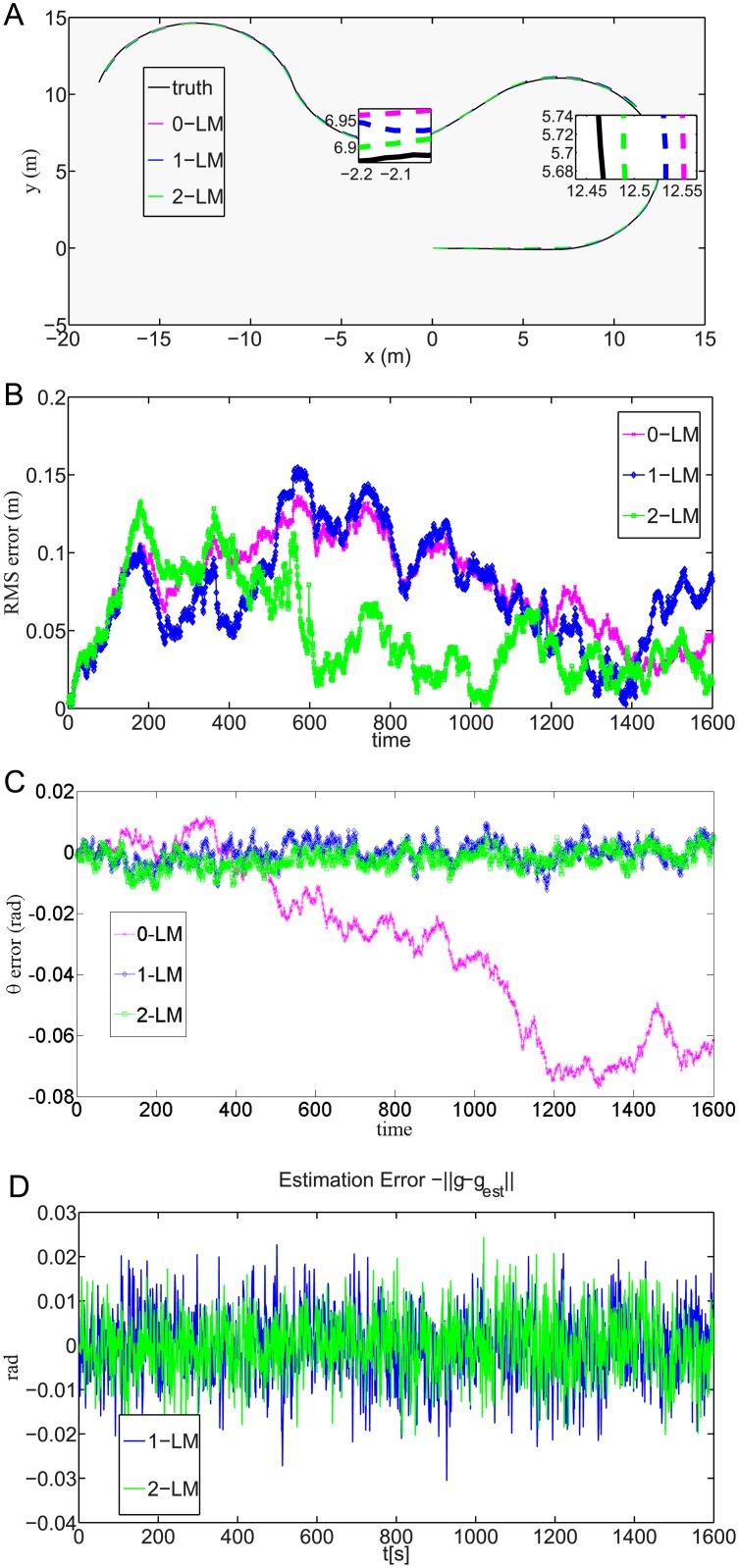
The leader robot system performance.

**Fig 6:**
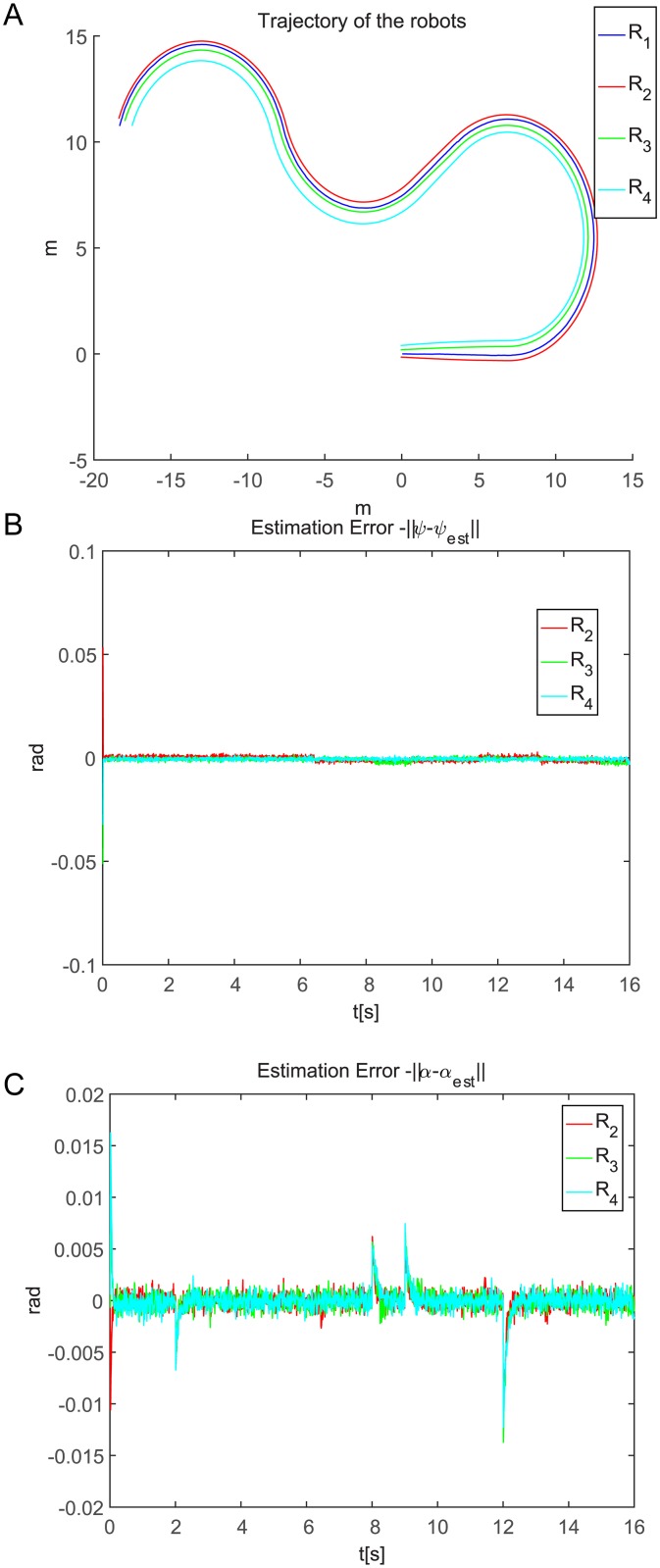
The leader-follower formation system performance.

This section presents the results of the simulation conducted to validate the observability conditions of the leader robot system and the leader-follower formation control discussed in the previous section. For the leader robot localization system, the simulation environment consists of one leader robot and two landmarks. For the leader-follower formation system, the simulation environment consists of one leader robot and three follower robots, where the followers (*R*_2_, *R*_3_, *R*_4_) follow the leader (*R*_1_). To demonstrate the validity of the proposed formation control approach, simulations are designed using both Webots 7 and Matlab. The simulation scenario given in Webots 7 is shown in Fig 4. The leader robot system performance and the leader-follower formation system performance are given in Figs 5 and 6, respectively. In Tables 1 and 2, the algorithmic (EIF versus PF) performances are given.

**Table 1:** Algorithm runtime: EIF Versus PF.

Algorithm	EIF		PF	
Landmark	one	two	one	two
Runtime (s)	0.013	0.013	0.199	0.186

**Table 2:** Algorithm performance: EIF Versus PF.

Algorithm	EIF		PF	
Landmark	one	two	one	two
γ Max Error	0.654	0.535	0.127	0.090
γ Max Error	0.024	0.020	0.006	0.005
θ Max Error	0.544	0.503	0.084	0.080
θ Max Error	0.014	0.019	0.004	0.004
X Max Error	0.511	0.469	0.495	0.457
X Max Error	0.068	0.012	0.018	0.024
Y Max Error	0.456	0.442	0.476	0.453
Y Max Error	0.051	0.049	0.051	0.048

### Initial conditions

In this study, we assume that the noise is white Gaussian. The simulation parameters that are used in this study are given as follows:

For PF, the sampling time is *T*_*c*_ = 0.01s; the sample particles are *N*_*t*_ = 500; the standard deviations of the robot state noise are ${\left[\begin{array}{ccc} \sigma_{x}{}_{{}_{i}} & \sigma_{y_{i}} & \sigma_{\theta_{i}} \end{array}\right]}^{\text{T}}={\left[\begin{array}{ccc} 0.05 & 0.05 & 0.05 \end{array}\right]}^{\text{T}}$; and the standard deviation of the measured noise is $\sigma_{\gamma }{}_{{}_{{}_{ik}}}=0.05$.

The parameters for the UKF and the input-output state feedback law are as follows:

We set ***s***(0) = [0.4474 2.4223 0 0.4360 2.4469 0 0.4126 1.816 0]^T^ and $s_{r}^{\text{ide}}={\left[\begin{array}{cccccc} 0.45 & 5\pi /4 & 0.45 & 5\pi /4 & 0.75 & 5\pi /4 \end{array}\right]}^{\text{T}}$, where the distances have units of meters and the angles have units of radians. The gains of the controller are *k*_1_ = 0.85 and *k*_2_ = 0.15. The other parameters of the UKF are *T*_*c*_ = 0.01s, *P*_*L*_ = *diag*([*h h*]), *P*_*N*_ = *diag*([*h h*]) and *P* = ([1.13 1.13]), where *h* = 3.0 × 10^−2^ rad^2^.

The following velocity inputs are assigned to the leader robot:

$$\begin{array}{l} v_{1}(t)=4m/\text{s}\\ \omega_{1}(t)=\{\begin{array}{c} \begin{array}{cc} 0 & \text{rad}/\text{s},t\in \left\{\left[0,2),(8,9\right]\right\} \end{array}\\ \begin{array}{cc} -\pi /5 & \text{rad}/\text{s},t\in [9,12] \end{array}\\ \begin{array}{cc} \pi /5 & \text{rad}/\text{s},t\in (12,16] \end{array} \end{array} \end{array}$$ (37)

The initial configuration vectors of the leader robot and the follower robots are

$$\begin{array}{l} {\left[\begin{array}{ccc} x_{1}(0) & y_{1}(0) & \theta_{1}(0) \end{array}\right]}^{\text{T}}={\left[\begin{array}{ccc} 0 & 0 & 0 \end{array}\right]}^{\text{T}}\\ {\left[\begin{array}{ccc} x_{2}(0) & y_{2}(0) & \theta_{2}(0) \end{array}\right]}^{\text{T}}={\left[-\begin{array}{ccc} 0.1 & -0.15 & 0 \end{array}\right]}^{\text{T}}\\ {\left[\begin{array}{ccc} x_{3}(0) & y_{3}(0) & \theta_{3}(0) \end{array}\right]}^{\text{T}}={\left[\begin{array}{ccc} -0.1 & 0.2 & 0 \end{array}\right]}^{\text{T}}\\ {\left[\begin{array}{ccc} x_{4}(0) & y_{4}(0) & \theta_{4}(0) \end{array}\right]}^{\text{T}}={\left[\begin{array}{ccc} -0.1 & 0.4 & 0 \end{array}\right]}^{\text{T}} \end{array}$$ (38)

In the initial moment, the robots must adjust their original positions to the ideal positions. The original position errors are relatively large, which has no effect on the normal movement of the robot formation. As a result, we do not consider the initial position errors when analyzing the simulations.

### Simulation analysis

Fig 5A shows the true and estimated trajectories of the leader robot for all three cases. The enlarged insets in Fig 5A show that the estimated trajectories with two different landmarks are those closest to the true trajectories. This is because with two different landmarks, the leader robot system is observable according to the observability condition described in Section 2.2. Fig 5B shows the root mean square errors with no landmark, one landmark and two landmarks. The root mean square errors with no landmark and one landmark are higher than those with two landmarks because the leader robot system is observable in the case with two landmarks. Fig 5C shows that the errors of *θ* for all three cases are very small. In addition, the errors for two landmarks are smaller than the errors for no landmark or one landmark because according to the observability condition described in Section 2.2, the leader robot system is observable with more than one landmark. Fig 5D shows the relative bearing errors for all three cases. Although the relative bearing errors for all three cases are small, the errors for two landmarks are smaller than the errors for one landmark.

Fig 6A shows the trajectories of the leader and the follower robots and shows that the desired formation is properly maintained. This is because the estimated trajectories of the leader robot are closest to the true trajectories when the leader robot system is observable. Fig 6B shows that the observation angle estimation errors are very small. Fig 6C shows that the direction angle estimation errors are also very small during the input-output feedback control process except when the leader suddenly adjusts the movement direction from right to left or from left to right; the maximum direction angle error occurs at approximately -0.015.

All of the simulation results indicate stable performance of the proposed formation control solution and a quick response to changes as long as the leader robot system is observable.

To conveniently compare the localization performance of the algorithms (EIF versus PF), we set *T*_*c*_ = 0.2*s* and *t* = 14*s* with the other initial conditions set as described above. Tables 1 and 2 show that the localization accuracy is higher for the PF than the EIF in [16]; however, the PF requires slightly more time to run than the EIF. In Table 2, the maximum errors of *γ* and *θ* greatly differ between the PF and the EIF, whereas the maximum errors of X and Y differ only slightly. The minimum errors of *γ*, *θ*, X and Y are approximately the same because all of the minimum errors are bounded.

## Conclusion

Based on the bearing-only observations, the nonlinear observability properties of the leader robot system and the leader-follower formation control are studied. When the leader robot system is observable, the leader-follower formation can be formed rapidly and then maintained. Simulation results are presented to demonstrate that the proposed approach can efficiently control the desired formation of multi-robots. Future research for multi-robot formation control should consider dynamic obstacles and formation transformation.

## Supporting information

S1 File The minimal data set. (RAR)
